# Changes in Out-of-Pocket Costs for US Hospital Admissions Between December and January Every Year

**DOI:** 10.1001/jamahealthforum.2023.0784

**Published:** 2023-05-05

**Authors:** Sneha Kannan, Zirui Song

**Affiliations:** 1 Harvard Combined Fellowship–Pulmonary/Critical Care, Massachusetts General Hospital and Beth Israel Deaconess Medical Center, Boston; 2 Department of Health Care Policy, Harvard Medical School, Boston, Massachusetts

## Abstract

This cross-sectional study examines cost-sharing for ICU and non-ICU hospitalizations among adults with employer-sponsored insurance.

## Introduction

Intensive care unit (ICU) admissions are generally unplanned. Given that ICU care is expensive and US patients face increasingly large deductibles that generally reset each calendar year, out-of-pocket costs for ICU care may be large at the beginning of the year.^1^ To date, little is known about cost-sharing for ICU admissions and cost-sharing changes from December to January, especially compared with non-ICU hospitalizations.^2^

## Methods

We examined 2010 to 2019 adult hospitalization data from MarketScan commercial database, which contains a large, nationwide convenience sample of commercially insured individuals. The Harvard Medical School Institutional Review Board approved this cross-sectional study. We followed the STROBE reporting guidelines.

We identified ICU admissions using revenue codes that indicated delivery of ICU services (eMethods in Supplement 1). For each hospitalization, we gathered age, sex, diagnostic cost group (DxCG) risk score, insurance plan type, metropolitan statistical area, diagnosis-related group (DRG), and paid amount. We decomposed cost-sharing into deductible, copayment, and coinsurance.

In unadjusted analyses, we calculated mean cost-sharing per ICU and non-ICU hospitalization by month of discharge among patients with high-deductible health plans (HDHPs) and other plan types (eMethods in Supplement 1). Hospitalizations spanning January 1 were excluded because cost-sharing may be assigned to multiple months. In adjusted analyses, we compared cost-sharing between December and January using an ordinary least-squares model adjusted for age, sex, DxCG risk score, hospital length of stay, metropolitan statistical area, and plan type, with fixed effects for each DRG and each December-to-January transition. The SEs were clustered by DRG.

Two-sided *P* < .05 indicated significance. Analyses were performed from October 1 to December 1, 2022, using Stata 16 (StataCorp LLC).

## Results

We identified 303 792 ICU and 2 043 619 non-ICU hospitalizations of commercially insured adults in December and January from 2010 to 2019. Among aggregate ICU hospitalizations, total cost-sharing averaged $1079 in December and $1871 in January, a 73.4% increase. Among non-ICU hospitalizations, total cost-sharing averaged $1043 in December and $1683 in January, a 61.3% increase (Figure). These increases and differences between ICU and non-ICU hospitalizations were larger among patients with HDHPs (Table). For patients with HDHPs requiring an ICU stay, cost-sharing averaged $3093 per hospitalization in January vs $1301 in December.

**Figure.**
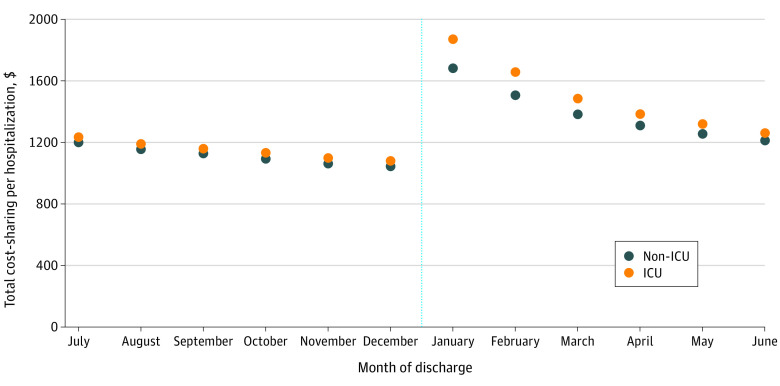
Aggregate Unadjusted Cost-Sharing per Hospitalization by Discharge Date All values were indexed to 2019 US dollars. The vertical line represents the transition to the new year. ICU indicates intensive care unit.

**Table.** Per-Hospitalization Cost-Sharing by Insurance Plan Type and ICU Admission Status^a^

	ICU hospitalization (n = 303 792)			Non-ICU hospitalization (n = 2 043 619)			Difference-in-difference between ICU and non-ICU hospitalizations	
	Unadjusted means in December, $	Unadjusted means in January, $	Adjusted difference, $ (95% CI)	Unadjusted means in December, $	Unadjusted means in January, $	Adjusted difference, $ (95% CI)	Adjusted difference, $ (95% CI)	*P* value
**HDHPs (n = 276 399)**								
Total cost-sharing	1301	3093	1822 (1729 to 1915)	1331	2951	1610 (1478 to 1743)	221 (75 to 367)	<.001
Coinsurance	898	1595	740 (672 to 807)	912	1299	411 (242 to 580)	321 (1501 to 492)	<.001
Deductible	389	1486	1081 (1044 to 1118)	399	1625	1191 (1109 to 1274)	−93 (−186 to 0)	.05
Copayment	14	12	1 (−4 to 5)	19	27	8 (2 to 14)	−8 (−14 to −1)	.02
**All other plans (n = 2 071 012)**								
Total cost-sharing	1051	1725	744 (698 to 789)	1003	1524	556 (482 to 629)	185 (103 to 266)	<.001
Coinsurance	749	1144	451 (415 to 487)	721	959	275 (202 to 348)	180 (102 to 258)	<.001
Deductible	216	476	271 (260 to 281)	198	467	268 (256 to 279)	−1 (−18 to 15)	.86
Copayment	87	104	22 (19 to 25)	83	98	13 (9 to 17)	6 (0 to 11)	.06

Abbreviations: HDHP, high-deductible health plan; ICU, intensive care unit.

^a^ Data were from 2010-2019 MarketScan commercial claims. Models were adjusted for age, sex, diagnostic cost group risk score, hospital length of stay, and metropolitan service area, with fixed effects by diagnosis-related group and each December-to-January transition. Values were indexed to 2019 US dollars.

While resetting of the deductible was a factor in the December-to-January cost-sharing increase among HDHPs, higher coinsurance explained the cost-sharing increase among other plan types. The Table shows that, across all plans, the ICU vs non-ICU differences in cost-sharing increase in January were associated with coinsurance. Results were nearly identical earlier (2009-2013) and later (2014-2019) in the study period.

## Discussion

Hospitalizations, especially ICU admissions, are often unpredictable. We found large cost-sharing increases annually from December to January 2010 to 2019 for both ICU and non-ICU hospitalizations, within the same DRGs, and adjusted for observable patient factors (as patients were newly exposed to higher out-of-pocket costs at the beginning of the year). These increases were larger for ICU vs non-ICU hospitalizations and for patients with HDHPs vs other plans. Over the course of 1 year, cost-sharing for hospitalizations decreased by more than 50%, depending on plan type and whether admission included an ICU stay.

These findings highlight a shortcoming of deductibles that are scheduled to reset at the beginning of the calendar year, which likely has unequal consequences for different patients. Patients with lower incomes and HDHPs, for example, are more likely to use acute care and least able to handle unexpected emergency expenses.^3,4^ Thus, these results resonate with recent reports of the benefits of resetting out-of-pocket costs or deductibles at shorter intervals throughout the year.^5,6^ Additionally, as the value of care commands greater attention from payers and policy makers, these findings underscore that inappropriate or low-value hospitalizations at the beginning of the year, particularly those involving ICU services, place a large financial burden on patients. A study limitation was the focus on hospitalizations of patients with employer-sponsored insurance; thus, the findings may not be generalizable to other populations.

## Notes

Supplement 1.
eMethods.

Supplement 2.
Data Sharing Statement
