# Supplementary material for: Changes in Out-of-Pocket Costs for US Hospital Admissions Between December and January Every Year
Source: JAMA Health Forum. 2023 May 5;4(5):e230784. doi: 10.1001/jamahealthforum.2023.0784 (PMC10163385; doi:10.1001/jamahealthforum.2023.0784)
Supplement: Supplement 1. — eMethods. [file jamahealthforum-e230784-s001.pdf]

Supplementary Online Content

Kannan S, Song Z. Changes in out-of-pocket costs for US hospital admissions between December and January every year. *JAMA Health Forum.* Published online May 5, 2023. doi:10.1001/jamahealthforum.2023.0784

eMethods.

This supplementary material has been provided by the authors to give readers additional information about their work.

© 2023 Kannan S et al. *JAMA Health Forum.*

eMethods

ICU Admission Classification

ICU admissions were identified by revenue code. Revenue codes 200, 201, 202, 203, 204, 205, 207, 208, 209, 210, 211, 212, 213, and 219 were used for ICU facilities in line with prior research.[*]

[*] Weissman GE, Hubbard RA, Kohn R, et al. Validation of an Administrative Definition of ICU Admission Using Revenue Center Codes. Crit Care Med. 2017;45(8):e758-e762.

Insurance classification

Within MarketScan, plan types 8 (Consumer-Driven Health Plan) and 9 (High Deductible Health Plan) were classified as “High Deductible”.[*]

[*] Truven Health Analytics. IBM MarketScan Research Databases. https://www.ibm.com/products/marketscan-research-databases/resources. Accessed April 2022.

© 2023 Kannan S et al. *JAMA Health Forum.*
