# Supplementary material for: Changes in Out-of-Pocket Costs for US Hospital Admissions Between December and January Every Year
Source: JAMA Health Forum. 2023 May 5;4(5):e230784. doi: 10.1001/jamahealthforum.2023.0784 (PMC10163385; doi:10.1001/jamahealthforum.2023.0784)
Supplement: Supplement 2. — Data Sharing Statement [file jamahealthforum-e230784-s002.pdf]

Data Sharing Statement

Kannan. Changes in Out-of-Pocket Costs for US Hospital Admissions Between December and January Every Year. *JAMA Health Forum*. Published May 05, 2023.
doi:10.1001/jamahealthforum.2023.0784

Data
Data available: No

Additional Information
Explanation for why data not available: The data come from the MarketScan databases - there is no original data collection. We will be happy to provide statistical analysis code/documentation on request.
